# Polyphenol-rich extracts enhance growth, immune function, and antioxidant defense in juvenile rainbow trout (*Oncorhynchus mykiss*)

**DOI:** 10.3389/fnut.2024.1487209

**Published:** 2024-12-05

**Authors:** Aghil Mansoori, Hamid Allaf Noveirian, Seyed Hossein Hoseinifar, Mirmasoud Sajjadi, Ghasem Ashouri, Roberta Imperatore, Marina Paolucci

**Affiliations:** ^1^Department of Fisheries, Faculty of Natural Resources, University of Guilan, Sowmeh Sara, Iran; ^2^Department of Fisheries, Faculty of Fisheries and Environmental Sciences, Gorgan University of Agricultural Sciences and Natural Resources, Gorgan, Iran; ^3^Department of Sciences and Technologies, University of Sannio, Benevento, Italy; ^4^National Artemia Research Center, Iranian Fisheries Science Research Institute, Agricultural Research, Education and Extension Organization (AREEO), Urmia, Iran

**Keywords:** polyphenols, oxidative stress, mucus, leukocyte, cytokine, rainbow trout

## Abstract

**Introduction:**

The present study was conducted to investigate the effects of PMIX, a polyphenol-rich extract mixture from chestnut wood and olive, on growth performance, hematological parameters, immunity in serum and skin mucus, hepatic antioxidant enzymes, and intestinal cytokine expression in rainbow trout (*Oncorhynchus mykiss*).

**Methods:**

Four experimental diets containing 0 g PMIX kg^−1^ diet (control, C), 0.5 g PMIX kg^−1^ diet (P0.5), 1 g PMIX kg^−1^ diet (P1), and 2 g PMIX kg^−1^ diet (P2) were fed to rainbow trout in an eight-week feeding trial. Triplicate groups of fish received each treatment. Growth performance, feed conversion ratio, protein efficiency, hepatosomatic and viscerosomatic indices, hematological parameters, immunity markers, hepatic antioxidant enzyme activities, and intestinal cytokine expression were determined.

**Results:**

PMIX supplementation significantly improved feed conversion ratios, while groups P0.5 and P1 evidenced an increase in growth performance along with protein efficiency ratios. It also showed decreased hepatosomatic and viscerosomatic indices in the P1 group. Except for higher percentages of monocytes in P0.5 and P1, most hematological parameters of the fish did not differ from that of the control. Serum lysozyme and respiratory burst activities were heightened in all PMIX-treated groups, and the skin mucus lysozyme activity was enhanced in P1. The blood phagocytic activity and phagocytic index, serum immunoglobulin, total protein, and bactericidal activity against *A. hydrophila* did not change. Hepatic superoxide dismutase activity significantly increased in P1 and P2, even though catalase activity did not change. Intestinal interleukin-6 expression was upregulated in all PMIX-treated groups, while tumor necrosis factor-alpha and interleukin-1 beta were upregulated in P1, P2, and P0.5 respectively.

**Discussion:**

The present results evidence that dietary polyphenols from chestnut wood and olive extracts enhance growth performance, antioxidant capacity, and several immunological parameters in the blood, skin mucus, and intestine of rainbow trout. A suitable supplementation level was 1 g PMIX kg^−1^ diet to separate these improvements.

## Introduction

Aquaculture is an important sector of the global food industry, providing a significant source of proteins for human consumption. According to the FAO report (1), for the first time, aquaculture with 94.4 million tonnes of aquatic animals, has surpassed capture fisheries, accounting for 51% of the total production. Freshwater fish accounts for 62% of total aquaculture production, with rainbow trout (*Oncorhynchus mykiss*) being one of the most farmed species worldwide (2).

The growing global demand for farmed fish calls for intensive culture conditions to meet the required production figures, although fish cultured under intensive programs are more susceptible to being victims of stress-related and infectious diseases (3, 4). Therefore, maintaining fish health and preventing disease outbreaks is crucial for the sustainability of aquaculture (84). Disease prevention and management strategies in aquaculture often involve the use of vaccines, antibiotics, and other pharmaceuticals, with negative impacts on the environment, as well as on fish and consumer health (5, 6). Consequently, alternative approaches, such as natural immunostimulants and health boosters, are being explored to enhance fish immunity and reduce the need for chemical treatments (7, 86).

Polyphenols are natural bioactive molecules produced by plants as secondary metabolites. In plants, polyphenols exert beneficial effects, among which the protection against infectious diseases transmitted by viruses, bacteria and fungi (8, 9). Due to their chemical structure based on one or more phenolic rings linked to hydroxyl groups, polyphenols possess an elevated antioxidant power which explains their health-promoting effects when assumed with the diet (85). In humans, polyphenols, other than antioxidants, have shown immunomodulatory, anti-inflammatory, antimicrobial, antitumor, anti-obesity and neuroprotective effects (10, 87). Research in recent years is refining knowledge on the countless actions of polyphenols, shedding light on the importance not only of the quantity administered but also their bioavailability and the synergistic effect of different polyphenols, which enhances their actions (11, 12).

Further, researchers have demonstrated that polyphenols are potent antioxidants, capable of combating inflammation and regulating the innate and adaptive immunity in fish (82, 83). Polyphenols extracted from chestnut shells have proven to be powerful intestinal anti-inflammatory molecules, providing beneficial effects in counteracting a pro-inflammatory diet and modulating the intestinal microbiome, thus mediating additional beneficial health effects in zebrafish (*Danio rerio*) (13, 14). The dietary supplementation of curcumin (15, 16), resveratrol (17), quercetin (18), and polyphenol-rich extracts from pomegranate peel (19), chestnut shell (20), and olive oilcake waste (21) improved the growth performance, antioxidant capacity, and immune responses in rainbow trout. Recent studies have demonstrated that co-administration of chestnut and olive polyphenols improves growth performance and antioxidant/immune responses in several fish species, including Asian seabass, *Lates calcarifer* (22), convict cichlid, *Amatitlania nigrofasciata* (23), beluga, *Huso huso* (24), and common carp, *Cyprinus carpio* (25), presenting this polyphenol mixture as suitable for aquaculture use.

Although there is unanimous agreement on the pro-health effects of polyphenols in aquaculture, the available evidence indicates that there are variations depending on the species, the phase of the life cycle and the doses used (88). Polyphenols may have variable effects on different fish species because they are absorbed in the fish intestine at different magnitudes (26); therefore, differences in intestinal characteristics (length, enzymes, microbiota, and evacuation time) can affect the overall responses of fish to dietary polyphenol concentrations (23). Therefore, the present study aimed to investigate the effects of the dietary mixture of chestnut and olive polyphenols (PMIX) on growth performance, hematological parameters, antioxidant activity, and immunological responses of rainbow trout juveniles.

## Materials and methods

### PMIX preparation

PMIX was prepared by mixing two commercial products, namely Silvafeed^®^ATX (Silvateam Co., Cuneo, Italy), which contained chestnut wood extract, and PhenoFeed^®^ (PhenoFarm Co., Rieti, Italy), contained olive extract. Silvafeed^®^ATX and PhenoFeed^®^ were mixed in a 9:1 ratio.

### Preparation of the diets

Commercial extruded feed (5 mm diameter) from Faradaneh Co. (Shahrekord, Iran) was used for feeding rainbow trout (*O. mykiss*). The chemical composition of the diet was as follows: crude protein 40.6%, crude lipid 14.5, crude ash 10.1%, moisture 7.9%, and total volatile nitrogen 0.71 mg g^−1^. A control diet without supplementation and three experimental diets supplemented by 0.5 g PMIX kg^−1^ diet (P05), 1 g PMIX kg^−1^ diet (P1), and 2 g PMIX kg^−1^ diet (P2) were prepared. PMIX was dissolved in dietary oil and sprayed on the feed pellets. Then, a 4% gelatin solution was used to coat the pellets before they were dried and stored in a refrigerator at 4°C. The control diet only received the gelatin solution coating.

The experiment involved 300 rainbow trout juveniles with an average weight of 60 g, distributed in 12 polyethylene round tanks with a volume of 300 L each. Each tank contained 25 fish, and the fish were acclimatized for 2 weeks before the start of the experiment. Each diet was offered to the fish of three tanks thrice a day until apparent satiation for 8 weeks. During the feeding trial, water dissolved oxygen, pH, temperature, and total ammonia were measured, being 7.2 ± 0.87 mg L^−1^, 7.65 ± 0.55, 16.0 ± 0.77°C, and 1.21 ± 0.33 mg L^−1^, respectively.

After the eight-week rearing period, the fish were weighed, and growth parameters were calculated as follows:

Weight gain (%) = 100× $\frac{\mathrm{Final weight}\;\left(g\right)-\mathrm{Initial weight}\;\left(g\right)}{\mathrm{Initial weight}\;\left(g\right)}$

Specific growth rate (SGR, %/day) = 100× $\frac{Ln\;\left(\mathrm{final weight}\right)-Ln\;\left(\mathrm{initial weight}\right)}{56}$

Feed conversion ratio (FCR) = $\frac{\mathrm{Feed intake}\;\left(g\right)}{\mathrm{Weight gain}\;\left(g\right)}$

Protein efficiency ratio (PER, %) = 100× $\frac{\mathrm{Weight gain}\;\left(g\right)}{\mathrm{Total dietary protein intake}\;\left(g\right)}$

Moreover, hepatosomatic (HSI) and viscerosomatic (VSI) indices were calculated as follows:

HSI (%) = 100 × $\frac{\mathrm{Hepatic weight}\;\left(g\right)}{\mathrm{Body weight}\;\left(g\right)}$

VSI (%) = 100 × $\frac{\mathrm{Visceral weight}\;\left(g\right)}{\mathrm{Body weight}\;\left(g\right)}$

### Sampling

At the end of the feeding trial, feeding was stopped for 24 h; then, three fish from each tank were caught and anesthetized using clove extract (0.5 g L^−1^). Blood was taken from the fish caudal vein using a syringe and differently processed. One sample was poured into a heparinized tube and used for respiratory burst (RB) activity assay, another sample was left to clot for 4 h at 4°C (27) and centrifuged at 2000 × *g* for 10 min at 4°C for serum separation, which was stored at −20°C until analysis. After the blood was taken, the fish were killed by hitting them on the head, and a piece of their liver was removed and frozen in liquid nitrogen for antioxidant parameters’ assays.

Another group of three fish from each tank was caught, anesthetized, and placed in a bag with 5 mL of 100 mM ammonium bicarbonate (NH_4_HCO_3_) H 7.8. After gently rubbing them for 1 min, an additional 5 mL of buffer was added, and the fish skin mucus sample was collected and centrifuged at 3000 × *g* and 4°C for 15 min, according to Ross et al. (28). The supernatant was collected and kept at −80°C until it could be analyzed. After collecting the skin mucus, the fish were killed as above and a piece of the posterior intestine was dissected and frozen in liquid nitrogen for molecular analysis.

### Serum/mucus lysozyme activity

The activity of lysozyme in serum and mucus was measured using a turbidimetric assay, as described by Ellis (29). This involved measuring the lytic activity of serum/mucus against lyophilized *Micrococcus luteus* by adding a specific volume of *M. luteus* (135 μL of *M. luteus* at a concentration of 0.2 mg mL^−1^ in 0.02 M sodium citrate buffer, pH 5.8) to serum or mucus samples and monitoring the change in optical density at 450 nm and 22°C. One unit of lysozyme activity was defined as the amount of serum/mucus causing a reduction in absorbance of 0.001 per min.

### Serum/mucus bactericidal activity

Bactericidal activities of serum and mucus were assessed according to Kajita et al. (30). This involved diluting serum and mucus samples in 0.1% gelatin-veronal buffer (containing calcium and magnesium ions, pH 7.5) and suspending *Aeromonas hydrophila* bacteria in the same buffer. The diluted serum or mucus and bacteria were mixed and incubated for 90 min at 25°C and cultured on TSA plates for 24 h to count the number of viable bacteria.

### Blood phagocytic activity, phagocytic index, and respiratory burst activity assay

To measure phagocytic activity, the method of Soltanian et al. (31) was followed with minor adjustments. Heparinized blood was used for the assay. Briefly, 1.9 × 10^7^ cells of *Staphylococcus aureus* in 0.1 mL of phosphate-buffered saline were added to 0.1 mL of blood samples in a microplate, mixed thoroughly, and incubated for 30 min. After incubation, the plate was gently mixed and smeared onto a glass slide. The smears were fixed in ethanol and stained with Giemsa (7%). Using microscopy, the phagocytic activity was determined by checking 100 phagocytic cells with engulfed bacteria. The phagocytic index was determined by averaging the number of the engulfed bacterial cells in ten phagocytic cells.

The RB activity assay was conducted by adding 100 μL of fresh blood to microtiter plate wells, followed by the addition of 0.2% nitroblue tetrazolium (NBT) solution to each well. The mixture was incubated at room temperature for 30 min. A sample of the NBT blood cell suspension (100 μL) was then combined with 2 mL of N, N-dimethyl formamide (Sigma, Germany) in a glass tube and centrifuged at 3,000 × *g* for 5 min. The supernatant was measured for optical density using a spectrophotometer at 620 nm, according to Abdelhamid et al. (32).

### Hematological analyses

The hematological analyses included measuring the hematocrit (Hct; %), hemoglobin concentration (Hb; g dL^−1^), and the number of red blood cells (RBC) and white blood cells (WBC), following the methods described by Blaxhall (33). Additionally, blood indices such as the mean corpuscular volume (MCV), mean corpuscular hemoglobin (MCH) and mean corpuscular hemoglobin concentration (MCHC) were calculated using the respective formulae after Blaxhall (33).

### Serum total protein and total immunoglobulin (Ig)

A commercial kit (Zistchem Co., Tehran, Iran) was utilized to determine the concentrations of total protein, following the manufacturer’s protocol. To estimate the total Ig in the serum, the Siwicki and Anderson (34) method was employed. A plastic vial containing 0.1 mL of serum was mixed with an equal volume of polyethylene glycol (concentration of 120 mg mL^−1^) and incubated at room temperature for 2 h with constant mixing. After centrifugation at 5,000 × *g* for 10 min, the supernatant was recovered, and protein concentration was determined. The total Ig concentration was calculated as the difference in total protein before and after the polyethylene glycol treatment.

### Hepatic antioxidant parameters

The hepatic samples were homogenized in 5 volumes (w:v) of ice-cold 100 mM phosphate buffer (KH_2_PO_4_), pH 7.4, containing 1.8% NaCl and 0.1 mM phenylmethylsulfonyl fluoride (PMSF) according to Regoli et al. (35). The homogenate was then centrifuged at 12000 × *g* for 15 min at 4°C (35), and the supernatant (excluding the top fat layer) was used to assess antioxidant parameters. Hepatic superoxide dismutase (SOD) activity was determined by measuring the reduction of NBT at 560 nm using a spectrophotometer (Biophotometer, Eppendorf, Germany) according to Fried (36). Hepatic catalase (CAT) activity was determined by measuring the decomposition of H_2_O_2_ at 240 nm, as described by Aebi (37). The concentration of soluble protein was determined in the hepatic extract using the Bradford method (38).

### Intestinal gene expression

The intestinal samples were analyzed for gene expression by extracting RNA using a commercial kit (RNeasy Mini Kit, Qiagen, Germany). To ensure that there was no DNA contamination, DNase I from Thermo Fisher Scientific was used to treat the samples. The quality of the final products was assessed using NanoDrop (2000c; Thermo Fisher Scientific) at 280/260 nm. The extracted RNA was then used to synthesize cDNA using a commercial kit from Thermo Fisher Scientific (USA; RevertAid™ First Strand cDNA). Specific primers were designed based on Table 1 to examine the expression of tumor necrosis factor-alpha (*TNF-α*), interleukin-1 beta (*il-1b*), and interleukin-6 (*il-6*) genes. Amplification of the cDNA was carried out using RT-PCR on a StepOne system (Applied Biosystems, Foster City, CA, USA). The reaction mixture contained 1 μL cDNA, 0.5 μL primer, and 5 μL SYBR Green (Amplicon A/S), which were brought up to 10 μL by adding diethylpyrocarbonate from Bio Basic Inc. (Bio Basic Inc., Ontario, Canada). The ∆∆Ct values were calculated for each treatment using *β-actin* as a housekeeping gene (R: GCAGACAGGTCCTCCACTA; F: ATGCAGAAGACAGCTACGTG), and the relative gene expression was calculated following the method described by Livak and Schmittgen (39).

**Table 1:** Sequences and accession numbers of primers.

Gene name	Abbreviations	Sequences of primers	Accession no	Annealing temperature (°C)	Function
Interleukin-1 beta	IL1ß	Forward: ACAGACATGGATTTTGAGTCA	AJ278242	59	Immunity
		Reverse: CTCATACTGTAATGTACTACTG			
Interleukin-6	*il-6*	Forward: ACTCCCTCTGTCACACACC	DQ866150	59	Immunity
		Reverse: GCAGACAGGTCCTCCACTA			
Tumor necrosis factor-alpha	TNF-α	Forward: CAAGAGTTTGAACCTCATTCAG	NM_001124374.1	59	Immunity
		Reverse: TGCACGATGCAGGACGGA			

### Statistical analysis

The data were analyzed by one-way ANOVA after being checked for normal distribution and homoscedasticity, using Shapiro–Wilk and Levene tests, respectively. Accordingly, SGR, HSI, VSI, and serum bactericidal activity were log-transformed before analysis. The percentile data were arcsine-transformed before analysis. Significant differences (*p* < 0.050) among the treatments were evaluated by the Duncan test. All data were analyzed in SPSS v. 22 and presented as mean ± SE.

## Results

Table 2 presents the growth performance and biometrical parameters. Final weight, weight gain, and PER significantly increased in the P1 and P2 treatments compared to the control. FCR and SGR significantly increased in the PMIX treatments, compared to the control, and the highest SGR was observed in the P1 and P2 treatments. HSI and VSI exhibited significant decreases in the P1 treatment compared to the control.

**Table 2:** Effects of dietary PMIX supplementation on growth performance and biometric parameters of rainbow trout (*Oncorhynchus mykiss*).

	C	P0.5	P1	P2	*p*-value
Initial weight (g)	89.07 ± 1.16	87.73 ± 0.53	88.00 ± 0.92	88.26 ± 0.27	0.681
Final weight (g)	223.06 ± 1.83a	223.76 ± 1.71a	231.50 ± 1.53b	230.03 ± 0.94b	0.010
Weight gain (%)	150.50 ± 2.49a	155.06 ± 1.18a	163.06 ± 1.27b	160.60 ± 0.32b	0.002
FCR	0.94 ± 0.02b	0.90 ± 0.01a	0.87 ± 0.01a	0.88 ± 0.01a	0.021
SGR (%/d)	1.64 ± 0.02a	1.67 ± 0.01b	1.72 ± 0.01c	1.71 ± 0.00c	0.001
PER	2.22 ± 0.05a	2.32 ± 0.02ab	2.39 ± 0.02b	2.37 ± 0.03b	0.024
HSI (%)	1.90 ± 0.12b	1.95 ± 0.02b	1.36 ± 0.10a	1.70 ± 0.01b	0.003
VSI (%)	17.65 ± 1.18b	15.76 ± 1.44ab	11.72 ± 0.40a	14.18 ± 1.43ab	0.041

C = 0 g PMIX kg^−1^ diet (control); P0.5 = 0.5 g PMIX kg^−1^ diet; P1 = 1 g PMIX kg^−1^ diet; P2 = 2 g PMIX kg^−1^ diet.

Different letters within a row indicate significant differences among the treatments.

Table 3 presents hematological parameters among the treatments. Dietary PMIX induced no significant changes in blood RBC, WBC, Hb, Hct, MCV, MCH, MCHC, lymphocyte percentage, neutrophil percentage, eosinophil percentage, and basophil percentage. Monocyte percentage significantly increased in the P0.5 and P1 treatments compared to the control.

**Table 3:** Effects of dietary PMIX supplementation on hematological parameters of rainbow trout (*Oncorhynchus mykiss*).

	C	P0.5	P1	P2	*p*-value
RBC (10^6^ μL^−1^)	1.51 ± 0.09	1.55 ± 0.12	1.58 ± 0.08	1.65 ± 0.06	0.759
Hct (%)	39.00 ± 0.86	40.5 ± 2.01	42.17 ± 1.28	41.76 ± 1.43	0.441
Hb (g dL^−1^)	11.32 ± 0.38	12.22 ± 0.43	12.15 ± 0.23	11.67 ± 0.36	0.261
MCV (fL)	262.00 ± 16.44	275.60 ± 18.79	270.40 ± 18.67	247.20 ± 15.23	0.679
MCH (pg)	76.59 ± 6.75	80.71 ± 5.43	77.74 ± 4.18	71.45 ± 4.52	0.669
MCHC (g dL^−1^)	29.0 ± 1.11	29.34 ± 0.51	28.93 ± 0.93	29.06 ± 1.29	0.993
WBC (10^3^ μL^−1^)	17.03 ± 0.97	16.43 ± 1.26	18.64 ± 0.93	19.76 ± 1.32	0.181
Monocyte (%)	3.83 ± 0.31a	4.83 ± 0.31b	5.50 ± 0.43b	4.67 ± 0.21ab	0.014
Neutrophil (%)	22.17 ± 2.03	20.67 ± 1.17	21.33 ± 1.45	21.83 ± 3.36	0.965
Lymphocyte (%)	70.76 ± 2.16	70.33 ± 1.45	69.17 ± 1.89	69.83 ± 3.57	0.973
Eosinophil (%)	2.17 ± 0.31	2.50 ± 0.43	2.83 ± 0.31	2.33 ± 0.49	0.670
Basophil (%)	1.17 ± 0.31	1.68 ± 0.21	1.17 ± 0.48	1.33 ± 0.33	0.710

C = 0 g PMIX kg^−1^ diet (control); P0.5 = 0.5 g PMIX kg^−1^ diet; P1 = 1 g PMIX kg^−1^ diet; P2 = 2 g PMIX kg^−1^ diet.

Different letters within a row indicate significant differences among the treatments (*n* = 6).

**Figure 1:**
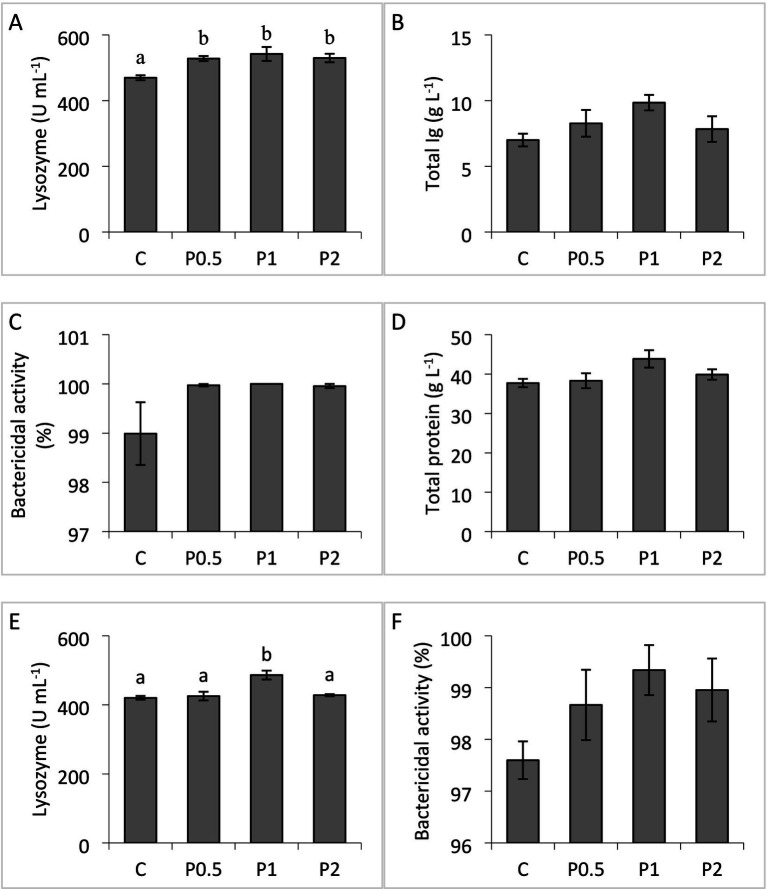
Effects of dietary PMIX supplementation on serum lysozyme **(A)**, total Ig **(B)**, bactericidal activity **(C)**, total protein **(D)**, skin mucus lysozyme **(E)**, and bactericidal activity **(F)** in rainbow trout (*Oncorhynchus mykiss*). C = 0 g PMIX kg^−1^ diet (control); P0.5 = 0.5 g PMIX kg^−1^ diet; P1 = 1 g PMIX kg^−1^ diet; P2 = 2 g PMIX kg^−1^ diet. Different letters above the bars indicate significant differences among the treatments (*n* = 6): serum lysozyme (*p* = 0.021), total Ig (*p* = 0.160), bactericidal activity (*p* = 0.144), total protein (*p* = 0.117), skin mucus lysozyme (*p* = 0.004), and bactericidal activity (*p* = 0.212).

Serum total protein and total Ig, and serum/mucus bactericidal activities exhibited no significant differences among the treatments. Dietary PMIX significantly affected lysozyme activity in the serum and skin mucus. Serum lysozyme activity significantly increased in the PMIX treatments, compared to the control; whereas mucus lysozyme activity significantly increased in the P1 treatment, compared to the other treatments (Figure 1).

**Figure 2:**
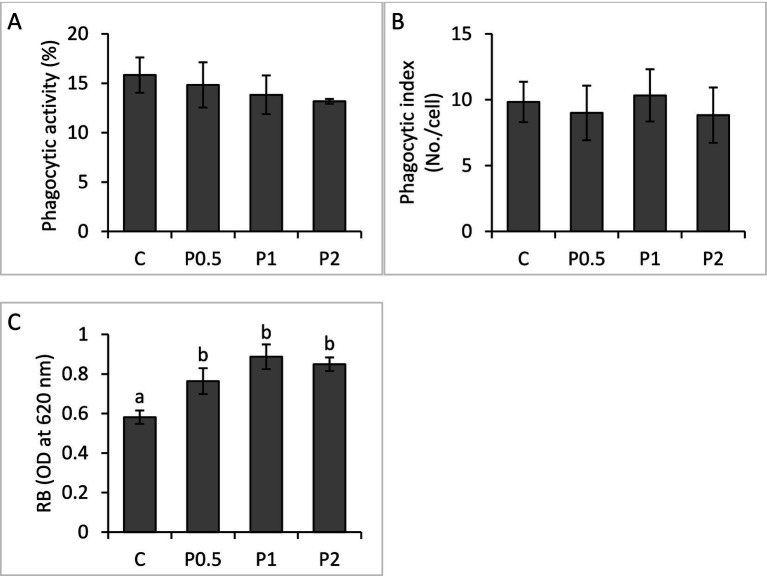
Effects of dietary PMIX supplementation on blood phagocytic activity **(A)**, phagocytic index **(B)**, and RB **(C)**, in rainbow trout (*Oncorhynchus mykiss*). C = 0 g PMIX kg^−1^ diet (control); P0.5 = 0.5 g PMIX kg^−1^ diet; P1 = 1 g PMIX kg^−1^ diet; P2 = 2 g PMIX kg^−1^ diet. Different letters above the bars indicate significant differences among the treatments (*n* = 6): phagocytic activity (*p* = 0.814), phagocytic index (*p* = 0.936), and RB (*p* = 0.012).

**Figure 3:**
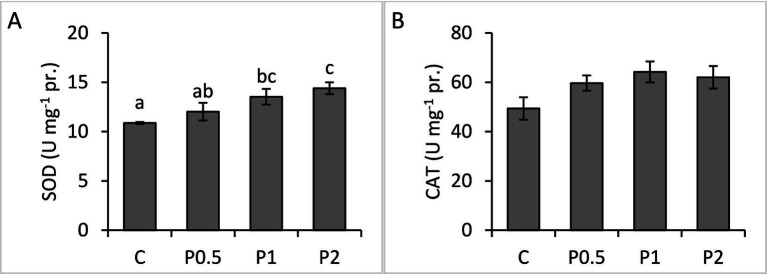
Effects of dietary PMIX supplementation on the hepatic SOD **(A)** and CAT **(B)** activities in rainbow trout (*Oncorhynchus mykiss*). C = 0 g PMIX kg^−1^ diet (control); P0.5 = 0.5 g PMIX kg^−1^ diet; P1 = 1 g PMIX kg^−1^ diet; P2 = 2 g PMIX kg^−1^ diet. Different letters above the bars indicate significant differences among the treatments (*n* = 6): SOD (*p* = 0.024), CAT (*p* = 0.134).

There were no significant differences in blood phagocytic activity and phagocytic index among the treatments; whereas RB activity significantly increased in the PMIX treatments, compared to the control (Figure 2). Hepatic SOD activity significantly increased in the P1 and P2 treatments, compared to the control; however, there were no significant effects of dietary PMIX on hepatic CAT activity (Figure 3).

**Figure 4:**
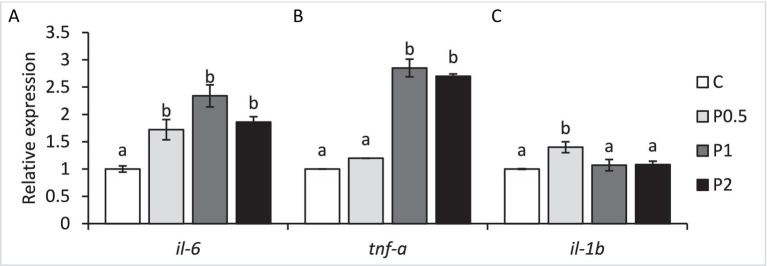
Effects of dietary PMIX supplementation on the intestinal *il-6* **(A)**, *tnf-α* **(B)**, and *il-1β* **(C)** expression (relative to *β-actin*) in rainbow trout (*Oncorhynchus mykiss*). C = 0 g PMIX kg^−1^ diet (control); P0.5 = 0.5 g PMIX kg^−1^ diet; P1 = 1 g PMIX kg^−1^ diet; P2 = 2 g PMIX kg^−1^ diet. Different letters above the bars indicate significant differences among the treatments (*n* = 6): *il-6* (*p* < 0.001), *tnf-α* (*p* < 0.001), *il-1β* (*p* = 0.003).

Intestinal *il-6* expression significantly increased in all PMIX treatments, whereas intestinal *tnf-α* exhibited up-regulations in the P1 and P2 treatments compared to the control. Intestinal *il-1β* expression significantly increased in the P1 treatment, compared to the control (Figure 4).

## Discussion

### Growth performance

Bioactive phytochemicals like polyphenols efficiently interact with proteins, DNA, immune cells, and other biological molecules to produce desired results (40). These compounds contain significant amounts of antioxidant activities. In the present study, PMIX improved growth performance and feed efficiency in rainbow trout (*O. mykiss*) by approximately 4–8%, while such improvements in common carp and beluga were ~ 25–26% and 25–32%, respectively. These differences are economically significant and affect fish performance under farming conditions. One important research pathway for polyphenol compounds is to clarify the final effect of functional foods and nutraceuticals enriched with them since the use of extracted polyphenol compounds presents differences and generated many controversies. Therefore, the effects of polyphenols on fish growth performance are diverse and species-dependent. For instance, the pomegranate peel has been found to have negative effects in common carp (41) but improves growth performance in rainbow trout (19). Moreover, resveratrol or curcumin may have positive (16, 17), negative (42), or no effects (43) on the growth performance of rainbow trout. Different factors such as fish species, fish size, experimental condition, dose, and duration of immunostimulants administration may affect the results (44). Nevertheless, polyphenols, when administered through the diet, have been observed to enhance the growth rate and feed efficiency in rainbow trout, as reported by several studies (15–17, 19, 21, 45, 46). Growth promotion by PMIX administration can be due to improvement of the intestinal health and/or the stimulation of the somatotropic axis. Supporting this, dietary administration of PMIX enhanced the growth performance in beluga (*H. huso*), accompanied by up-regulation of the growth and insulin growth factor-1 genes expression (24). Safari et al. (24) suggested that the effects of the PMIX on fish growth performance depend on the species. In Asian seabass (*L. calcalifer*), a 2.5 g PMIX kg^−1^ diet increased growth rate but not feed efficiency after 6 weeks, while other doses were ineffective (22). Beluga (24) and common carp, (*C. carpio*) (25) exhibited improvements in growth rate and feed efficiency when fed diets containing 0.5–2 g PMIX kg^−1^ diet; however, the magnitude of these improvements differs from the present study.

The use of high-fat diets in modern aquaculture has become increasingly common due to the fat potential benefits in terms of growth performance and feed efficiency. However, high-fat diets can also lead to negative effects on fish health and welfare, such as decreased immune function and increased susceptibility to diseases (47). Polyphenols have been studied as potential additives to high-fat diets to mitigate these negative effects (48). The present results suggest that a 1 g PMIX kg^−1^ diet can reduce fat deposition in rainbow trout, characterized by lower VSI and HSI values. In this regard, goldfish exhibited lower fat deposition and attenuated fatty liver disease induced by a high-fat diet, when treated with polyphenols extracted from olive mill waste water (49). There is no study regarding the role of chestnut polyphenols on fat metabolism in fish; however, a study on mice reported that chestnut polyphenols inhibit lipid synthesis and increase fatty acid oxidation in animals fed on a high-fat diet (50). A previous study carried out on Asian seabass showed that dietary PMIX supplementation increased HSI (22); hence further studies on lipid metabolism pathways are necessary to exactly address the mode of action of PMIX in fish.

### Hematological parameters

Hematological parameters are useful means to assess fish welfare, as biomarkers of stress, nutritional status, and environmental pollution. The hematological parameters of fish are observed to be affected by a variety of factors, which include species, size, age, physiological status, environmental conditions, and diet, e.g., quality and quantity of food, dietary ingredients, protein sources, vitamins, additives (51). The present study shows that PMIX had no such effects in rainbow trout, which is in line with the findings in convict cichlid (23), beluga (24), and common carp (25), fed PMIX-supplemented diets. On the other hand, Asian seabass exhibited significant elevations in these parameters, following PMIX administration (22), suggesting possible intra-species differences regarding hematological responses to PMIX administration in fish.

### Immune responses

Leukocytes are important immune cells residing in the blood, but able to migrate to infected tissues to eliminate foreign germs (52). The present results are in line with those obtained in Asian seabass (22), where PMIX induced no significant change in WBC count. Besides, the present study shows that PMIX could increase monocyte percentage in rainbow trout. Safari et al. (24) demonstrated that the WBC differential count was not affected by PMIX treatment in beluga. Monocytes are a type of WBC that play a key role in the immune system, helping to identify and eliminate harmful invaders. When fish are exposed to pathogens, monocytes activate and begin to produce various immune molecules, such as cytokines and antibodies, which help to fight off the infection (53). Research studies have shown that certain dietary herbal additives can increase monocyte percentages in fish (52, 54, 55). Moreover, it has been shown that the increase in monocyte number after dietary supplementation of herbal additives is accompanied by higher survival after bacterial challenges (56, 57).

It has been reported that several cell types possess polyphenol receptors that can modulate signaling pathways to involve immune responses (89). Phagocytosis in vertebrates has been recognized as a critical component of innate and adaptive immune responses to pathogens (58). Phagocytosis is a vital process for uptaking and destructing the microbes, as well as for initiating and developing adaptive immune responses (58). Phagocytes, including monocytes/macrophages and granulocytes, are essential in processing pathogens and producing antibodies (59). Phagocytosis is well known to elicit several antimicrobial mechanisms, and among them, the most important one is the production of reactive oxygen and nitrogen intermediates. The rate of phagocytosis is determined by various cell-related factors, such as the pathogen recognition receptors (PRRs) (58). Based on present results, it appears that PMIX failed to stimulate phagocytic activity in rainbow trout, however, RB outputs suggest that PMIX administration increases reactive oxygen species production by phagocytes. This increase in RB activity may be due to the higher antioxidant capacity of the phagocytes. Respiratory burst and phagocytosis seem to be differently modulated by polyphenols depending upon their structure, concentration, way of administration, cellular localization, and concentration (60, 61). Coccia et al. (20) revealed that the phagocytic activity was decreased with low doses of chestnut shell extract and increased with high doses in both blood and intestinal leukocytes of rainbow trout. Although the present study did not examine the antioxidant capacities of the phagocytes or whole blood, dietary administration of PMIX showed strong antioxidant activity and stimulate hepatic SOD in this study. In agreement with our findings, it has been demonstrated that supplementing the diet with PMIX enhanced the antioxidant capacity in Asian seabass (22), beluga (24), and convict cichlid (23). Additionally, low antioxidant capacity limits RB activity in fish (59). Therefore, PMIX potentially could be able to improve RB activity in rainbow trout by enhancing antioxidant capacity. This enhancement in RB activity may improve disease resistance in rainbow trout, as reported in Nile tilapia fed with chestnut polyphenols (62).

Lysozyme is a crucial component in the immune system of fish. It plays an important role in their immune system as it is bactericidal by hydrolyzing bacterial cell wall peptidoglycans, resulting in bacteriolysis (63). It is also known to act as an opsonin (64). Besides, lysozyme stimulates phagocytosis of the granulocytes (65). In the present study, the serum lysozyme activity was enhanced in all the PMIX treatment groups. It has been well-documented that dietary olive wastes (21), chestnut polyphenols (62), or PMIX (22–25) improve serum/mucus lysozyme activity in different fish species. An increase in lysozyme activity is considered an indicator of boosted immune strength, as it helps the host eliminate invading bacteria at early stages (66, 67). On the other hand, improvement in the skin mucus lysozyme activity may be effective in the protection of rainbow trout against disease in farms, as surrounding water is the main route of pathogen transmission (68).

### Antioxidant enzymes

Polyphenols are well-known for their antioxidant effects and several studies have demonstrated that the antioxidant capacity of fish is improved by dietary polyphenol administration (48). The antioxidant effect of polyphenol compounds essentially consists of scavenging free superoxide and hydroxyl radicals by donating a proton of a hydroxyl group attached to the aromatic ring and thus preventing high levels of reactive oxygen species, reactive nitrogen species, and oxidation of sensitive biomolecules like proteins or lipids (69). Superoxide dismutase is an important antioxidant enzyme in the electron transfer chain, where it neutralizes superoxide molecules and protects the cell against oxidative damage (70). Hepatocytes contain a large number of mitochondria; thus, by boosting hepatic SOD, PMIX can be hepatoprotective. Similar elevation in the SOD activity has been observed in rainbow trout fed with olive wastes supplementation diets (21) and in beluga fed with PMIX (24). Catalase involves scavenging superoxide anions which are produced during the respiratory burst of phagocytes (71). In the present study, hepatic CAT was not affected by different dietary PMIX treatments. Similarly, hepatic CAT activity has not changed in Asian seabass fed with diets supplemented by PMIX up to 2.5 g kg^−1^ diet (22).

### Intestinal cytokines

The fish intestine is an immune-involved system (as a primary defense barrier) and has various immune-related components such as lysozyme, alkaline phosphatase, Ig, protease, and complement proteins (72). Besides, due to constant contact with the surrounding water, the immune status of the fish intestine (particularly the posterior part) is very important in disease prevention (73). Cytokines are important immune-related molecules regulated by dietary, microbial, and environmental factors (74–76). The anti-inflammatory effect of polyphenol compounds primarily consists of interfering with immune cell regulation proinflammatory cytokine (IL-6, IL-8) synthesis as well as the aforementioned scavenging of nitric oxide radicals produced by macrophages (77). Fish *tnf-α* family members may play a role in regulating leukocyte homing, proliferation, and migration, while *il-1β* and *il-6* are involved in the activation and proliferation of lymphocyte and phagocyte cells (78). Multiple studies have suggested that adding various supplements to fish diets can lead to increased expressions of pro-inflammatory cytokines, including *tnf-α*, *il-1β*, and *il-6* in the intestines, which have been linked to enhanced disease resistance (79, 80, 90). Effects of dietary olive or chestnut polyphenols on intestinal cytokines have been variable in fish. An *in vitro* analysis of rainbow trout intestinal leukocytes has revealed that chestnut shell extract up-regulates expression of *tnf-α* and *il-1β* (20). Dietary olive waste has significantly up-regulated *tnf-α*, and *interleukin-8*, but not *il-1β* expressions in the intestine of rainbow trout (21). Olive leaf extract has also up-regulated intestinal *tnf-α* and *il-1β* expressions in common carp (73). On the other hand, polyphenols extracted from the chestnut (*Castanea sativa*) shell, agri-food waste rich in tannins, and mullein (*Verbascum macrurum*), a perennial spontaneous plant rich in flavonoids, have had no significant effects on pro-inflammatory cytokines’ expression in the intestine of zebrafish, *Danio rerio* (81). The findings of this study showed that PMIX could be of some utility in designing functional diets and feeding schedules for farmed fish.

## Conclusion

In conclusion, the results of this study demonstrate that PMIX can improve growth performance, modulate immunological responses in the blood, skin mucus, and intestine of rainbow trout and maintain the anti-inflammatory role exercised by these bioactive molecules in fish. The main strength of the present work is the preparation of a feed additive from waste that can beneficially affect trout. However, determination of the best polyphenols and their mixture for trout aquaculture requires additional studies. Overall, based on the present study, a 1 g PMIX kg^−1^ diet is recommended for rainbow trout feed supplementation.

## Data Availability

The original contributions presented in the study are included in the article/supplementary material, further inquiries can be directed to the corresponding author.
